# Complete Renal Artery Embolization in a Comorbid Patient with an Arteriovenous Malformation

**DOI:** 10.1155/2014/856059

**Published:** 2014-03-04

**Authors:** Anthony Thayaparan, Tarik Amer, Eamon Mahdi, Omar Aboumarzouk, Owen Hughes

**Affiliations:** Urology Department, University Hospital Wales, Cardiff, UK

## Abstract

Renal arteriovenous malformations are uncommon and are rarely a cause for presentation. However, they can occasionally pose life-threatening problems. We present a case of an elderly female who underwent a complete left renal artery embolization, following a difficult presentation complicated by advanced dementia and multiple comorbidities. This is the first documented case of complete renal artery embolisation used to treat a renal AVM.

## 1. Introduction

Renal arteriovenous malformations (AVMs) are aberrant connections between the renal artery and vein. They may be acquired or congenital in their aetiology [1]. The most common presenting complaint is of visible haematuria. It is AS part of the routine workup for frank haematuria that intrarenal AVMs are diagnosed [2]. Other presentations include hypertension of unknown cause, abdominal pain, and left ventricular hypertrophy [1, 2].

In cases where patients are unstable requiring intervention, the historical treatment has been partial or total nephrectomy with arterial reconstruction [2]. Over time, the range of options has evolved to include conservative and angiographic procedures [2]. We present the only documented case of complete renal artery ablation being used in the acute setting.

## 2. Case Presentation

A frail 76-year-old lady with vascular dementia presented with a five-week history of malaise and persistent gross haematuria. Other than dysuria she had no other lower urinary tract symptoms. Her past medical history consisted of chronic hyponatraemia, advanced vascular dementia, osteoarthritis, and glaucoma. On admission, she looked unwell and had pallor but was haemodynamically stable. Her abdomen was soft and nontender, her lung fields were clear, and her heart sounds normal. Her haemoglobin was 11.9 g/dL and WBC was 7.6 × 10^9^/L, and she had a grossly normal renal function with urea of 13.4. Her CRP was not performed. The initial working diagnosis was of a urinary tract infection and she was commenced on oral coamoxiclav. She responded well initially but soon deteriorated passing further frank blood necessitating blood transfusion. As part of her haematuria workup, a renal tract ultrasound was performed. This showed a calcific density in the right kidney whilst the left appeared normal. The bladder was noted to contain an adherent mass consistent with clots. A 3-way catheter was sited and regular washouts and irrigation were instated. After passing old clots, the urine promptly cleared but the following day she became septic and was treated aggressively with fluid resuscitation and intravenous coamoxiclav and gentamicin. She experienced further large quantities of haematuria requiring multiple blood transfusions.

**Figure 1:**
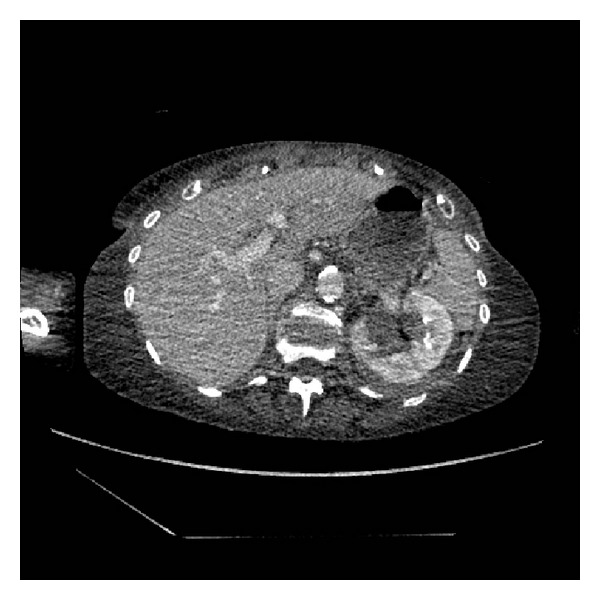
Split bolus contrast CT KUB: transverse section, left kidney.

**Figure 2:**
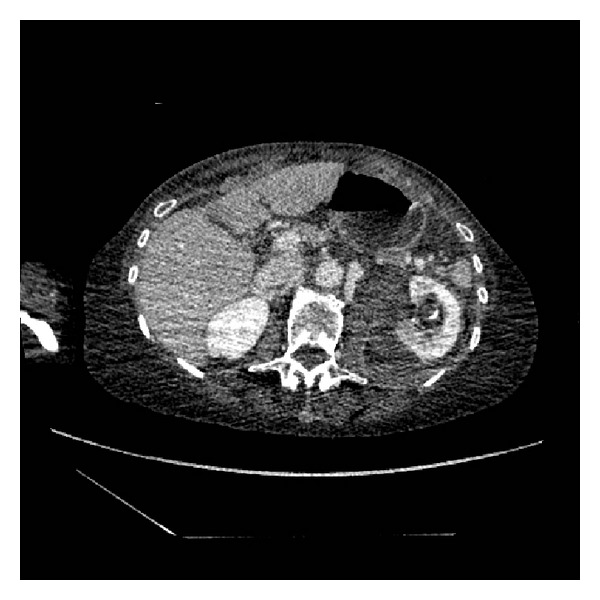
Split bolus contrast CT KUB: transverse section, left kidney.

**Figure 3:**
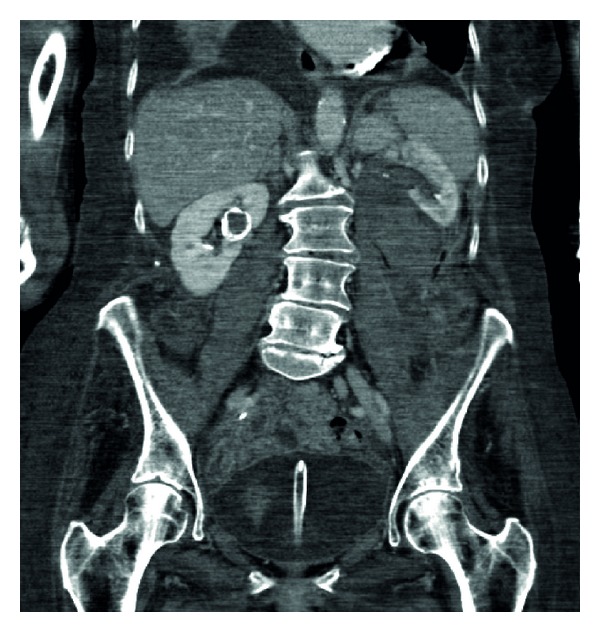
Split bolus contrast CT KUB: coronal section, left kidney with hydronephrosis and right kidney calcification.

**Figure 4:**
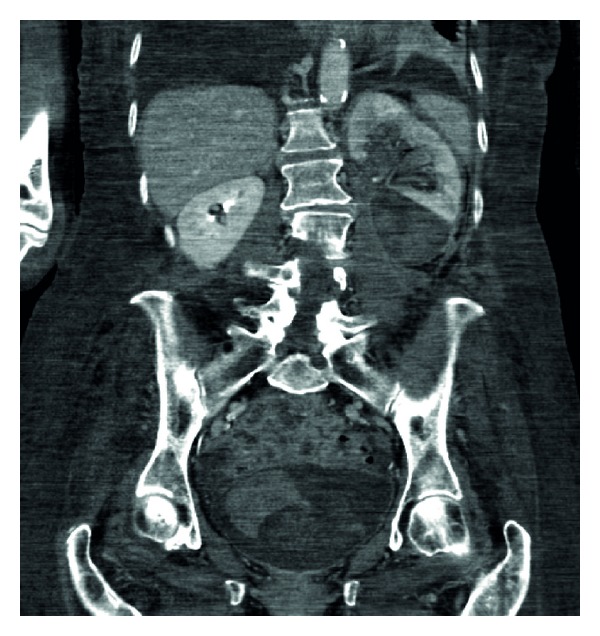
Split bolus contrast CT KUB: coronal section.

**Figure 5:**
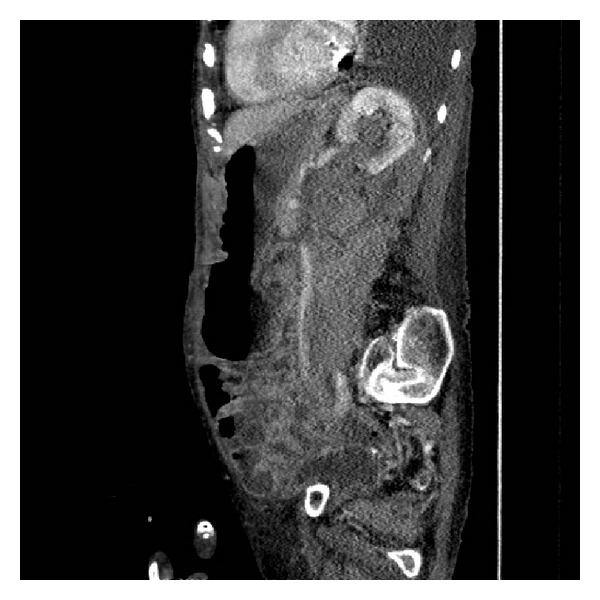
Split bolus contrast CT KUB: sagittal section.

She went on to have a CT urogram. This showed a mild left sided hydronephrosis and fluid of a higher density than urine in the lower calyces presumed to be a clot (Figures 1 and 2). A split bolus contrast study subsequently showed left pelvicalyceal dilatation with perinephric inflammatory changes and high attenuation changes of fat. The left ureter almost certainly contained blood and a possible tumour (Figures 3, 4, and 5).

**Figure 6:**
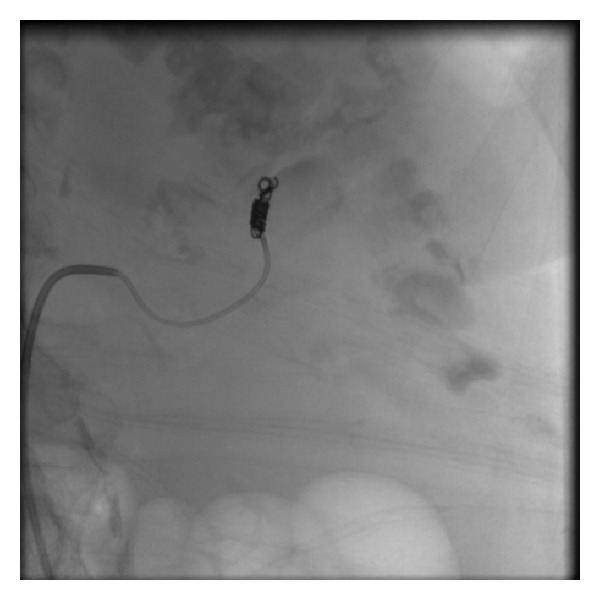
Left renal angiography and embolisation. Coil seen. Note also the accessory renal artery occluded with occlusive glue (Glubran), not visualised.

Due to her comorbidities, she was deemed unfit for surgical intervention. A multidisciplinary team meeting concluded that renal angiography and complete embolization were the most suitable option to achieve haemostasis (Figure 6). Accordingly and successfully, the left renal artery was completely embolised. The haematuria resolved and the urine remained clear for the rest of the admission.

Several days later, the intervention was complicated by sepsis secondary to a presumed necrotic organ. With antimicrobial therapy she made a remarkable recovery and was deemed fit for discharge two weeks later.

Interestingly, the cause of bleeding was not found until the images of the angiogram were reviewed again after intervention. These showed a bleeding arteriovenous malformation.

## 3. Discussion

Acquired renal AVMs or arteriovenous fistulae are sometimes iatrogenic in origin following traumatic renal biopsies, partial nephrectomy, and ureteroscopy [1–3]. Renal carcinoma, owing to its propensity to follow renal veins [4], and idiopathic causes [2, 4] are other documented causes of this fortunately rare condition. There are only 200 cases of congenital AVMs in the literature. The two noteworthy types of congenital renal AVMs are cirsoid and the less frequently occurring cavernous congenital. These account for less than 30% of cases [2]. Cirsoid AVMs are large with multiple feeder vessels. Acquired AVMs are more often aneurysmal in nature and it is these which produce cardiovascular features. Usually, renal AVMs do not warrant intervention unless in the presence of persistent haematuria, hypertension, or heart failure [5].

The typical presentation of this condition is macroscopic haematuria and rarely brittle hypertension [6]. Hypertension occurs due to a relative hypoperfusion distal to the AVM causing increased renin secretion. Although ultrasound and CT can detect intrarenal AVMs [7, 8], the gold standard for diagnosing a renal AVM is renal angiography [9]. This corroborates our experience where the AVM was detected only at angiography. Typically, the radiologist will detect a rapid movement of contrast from the arterial system to the inferior vena cava due to the AVM or a subtle blush [9].

Treatment is surgical or angiographic [1]. Angiographic embolisation is being used with increasing frequency as it is minimally invasive, has fewer complications [5], and preserves renal mass [10, 11] versus partial or complete nephrectomy. Materials used for embolization range from steel coils, gelatin sponges, foams, and synthetic polymers to autologous blood clots and alcohol [10, 11].

This is the first case where a patient who clinically required a nephrectomy was treated with this minimally invasive technique to achieve renal infarction and thus haemostasis.

## 4. Conclusion

This case highlights two points. Firstly, do not forget the differential diagnosis of renal AVM's in patients with persistent haematuria with undetermined abnormalities in the upper urinary tract. Secondly, as this case shows, forward planning by urologists and radiologists could lead to decisions of complete renal embolisation if clinically necessary. This presents an alternative to major surgery, particularly in the elderly and comorbid populations.
